# Stringently regulated p23 expression is critical for coordinated movement in mice: implications for Alzheimer’s disease

**DOI:** 10.1186/1750-1326-7-S1-L2

**Published:** 2012-02-07

**Authors:** Ping Gong, Jelita Roseman, Kulandaivelu S Vetrivel, Vytautas P Bindokas, Lois A Zitzow, Satyabrata Kar, Angèle T Parent, Gopal Thinakaran

**Affiliations:** 1 Department of Neurobiology, The University of Chicago, Chicago, Illinois, USA; 2 Department of Neurobiology, Pharmacology, and Physiology, The University of Chicago, Chicago, Illinois, USA; 3 Department of Surgery, The University of Chicago, Chicago, Illinois, USA; 4 Departments of Medicine and Psychiatry, Centre for Prions and Protein Folding Diseases, University of Alberta, Edmonton, Alberta, Canada

## Background

p23 belongs to the highly conserved p24 family of type I transmembrane proteins, which participate in the bidirectional protein transport between the endoplasmic reticulum and Golgi apparatus. The mammalian p23 has been reported to interact with γ-secretase complex, the enzyme responsible for intramembrane proteolysis of amyloid precursor protein (APP) and other substrates. In cultured cells knockdown of p23 expression increases secretory trafficking as well as γ-secretase processing of APP to release higher levels of APP ectodomain and β-amyloid peptides into the medium. Interestingly, in mouse and human brain the steady-state levels of p23 decline during aging. Together, these two lines of evidence suggest that elevation of p23 expression in adult neurons might mitigate cerebral amyloid burden.

## Results

We generated several lines of transgenic mice expressing human p23 in neurons under the control of *Thy-1.2* promoter. We found that even a 50% increase in p23 levels in the central nervous system of mice causes post-natal growth retardation, severe neurological problems characterized by tremors, seizure, ataxia, and uncoordinated movements, and premature death. The severity of the phenotype closely correlated with the level of p23 overexpression in multiple transgenic lines. While the number and general morphology of neurons in Hup23 mice appeared to be normal throughout the brain, abnormal non-Golgi p23 localization was observed in a subset of neurons with high transgene expression in brainstem. Moreover, detailed immunofluorescence analysis revealed marked proliferation of astrocytes, activation of microglia, and thinning of myelinated bundles in brainstem of Hup23 mice. These cellular responses occurred concomitantly with the activation of the unfolded protein response in brainstem of several lines of Hup23 mice.

## Conclusion

These results demonstrate that proper levels of p23 expression is critical for neuronal function, and perturbing p23 function by overexpression initiates a cascade of cellular reactions in brainstem that leads to severe motor deficits and other neurological problems, which culminate in premature death. The neurological phenotype observed in Hup23 mice has significant implications for translational Alzheimer’s disease research aimed at stimulating p23 expression or function with the goal of reducing cerebral amyloid burden via negative modulation of γ-secretase. Moreover, our report has broader relevance to molecular mechanisms in several neurodegenerative diseases as it highlights the inherent vulnerability of the early secretory pathway mechanisms that ensure proteostasis in neurons.

